# Sigmoid colon pseudotumor of actinomycosis: a rare case

**DOI:** 10.1093/jscr/rjad697

**Published:** 2024-01-04

**Authors:** Andy Lesmana, Vika Wirdhani, Lenti B R Perangin Angin, Muhamad I Muzakky, Stefi G V Hayon, Mentari M Sholihah, Muhammad I Hanif

**Affiliations:** Surgery Department, Dr. Sitanala Central Hospital, Tangerang, Indonesia; Internal Medicine Department, Dr. Sitanala Central Hospital, Tangerang, Indonesia; Pathology Department, Dr. Sitanala Central Hospital, Tangerang, Indonesia; Emergency Department, Dr. Sitanala Central Hospital, Tangerang, Indonesia; Emergency Department, Dr. Sitanala Central Hospital, Tangerang, Indonesia; Emergency Department, Dr. Sitanala Central Hospital, Tangerang, Indonesia; Emergency Department, Badau Primary Health Care, Kapuas Hulu, Indonesia

**Keywords:** actinomycosis, pseudotumor, sigmoid colon, rare case

## Abstract

Actinomycosis is a chronic suppurative infection caused by gram-positive bacteria, Actinomyces, which commonly colonize the oral cavity, urogenital tract and gastrointestinal tract. It causes opportunistic infection where the mucosal barrier is compromised and is often misdiagnosed as malignancy. We report a case of a 58-year-old female with severe abdominal pain and a palpable tender mass in the left lower quadrant. Computed tomography scan with contrast showed thickening of the transverse, descending to sigmoid colon wall and intense contrast enhancement resembling colitis with mesenteritis. At laparotomy, we found an adherent mass involving sigmoid colon with adjacent small bowel and peritoneum. We decided to perform adhesiolysis and Hartmann procedure. The culture result was negative, whereas the biopsy of sigmoid colon revealed characteristic sulfur granules of actinomycosis colony. Intravenous antibiotic combination of ceftazidime and metronidazole was administered for 14 days followed by complete resolution of symptoms. Histopathological and bacteriological examinations are keys to diagnose actinomycosis. Patients require long-term antibiotic therapy, but surgery is often required because preoperative diagnosis is difficult.

**Main novel aspects?:**

- We presented the rare case of intra-abdominal actinomycosis in Indonesia
- The case was challenging to diagnose pre-operatively based on physical examination and
- Exploratory laparotomy revealed pseudotumor mimicking advanced stage of colon neoplasm

## Introduction

Actinomycosis is a chronic suppurative infectious disease caused by Gram-positive *Actinomyces israelii* organisms [1]. In physiologic condition, this non-spore forming anaerobic bacterium is found in the mucosa of the oral cavity and upper gastrointestinal tract [1]. It became pathogenic only in the presence of damaged mucosal barriers [1, 2]. Preoperative diagnosis may be difficult due to non-specific clinical presentation and varying radiological findings. The diagnosis is mostly based on histopathological finding after surgical biopsy and rarely identified by tissue culture [3]. Histopathologic findings show necrosis with yellowish sulfur granules and filamentous Gram-positive fungal-like pathogen [3, 4]. Abdominal actinomycosis is commonly located in appendix, cecum, and colon. The sign and symptoms that appear related to its anatomical site and mostly manifested as abdominal pain with palpable mass or an inflammatory pseudotumor [3]. Based on a review study of actinomycosis cases, most cases were in the age group of 30–60 years old and male is more common than female. The risk factor for mucosal damage is related to previous surgery or intrauterine device. From an analysis study of abdominal actinomycosis, 84% of cases had history of emergency surgical procedures for acute gastrointestinal lesions prior to the onset of the actinomycosis [3, 5].

Here, we report a rare case of pathologically confirmed abdominal actinomycosis that was preoperatively considered as intestinal neoplasm with acute abdominal pain.

## Case presentation

**Figure 1:**
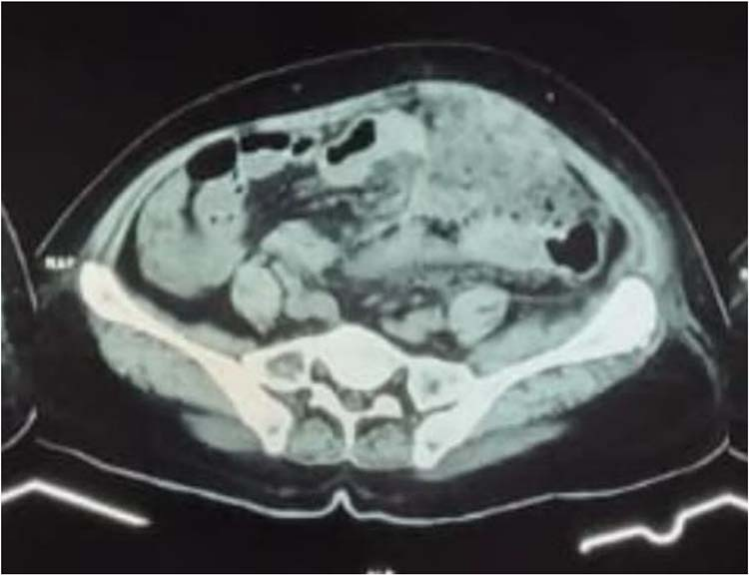
Abdominal CT scan with contrast showed transverse, descending to sigmoid colon wall thickening and significant contrast enhancement supporting colitis with mesenteritis.

A 58-year-old female came to our emergency department with left-lower quadrant abdominal pain since 5 days before admission without nausea and vomiting. Three weeks before admission, she had history of urinary retention and constipation without history of fever. On physical examination, abdominal tenderness associated with a palpable mass was found on the left lower quadrant. The mass was ~15 cm in diameter. Her laboratory result showed increased level of white blood cells. We later performed abdominal X-ray, ultrasound (USG), and computed tomography scan (CT scan). The X-ray showed local obstruction in the right lower abdomen and USG revealed a left abdominal colon mass. For further evaluation, we conducted abdominal CT scan with contrast that showed thickening of the transverse, descending to sigmoid colon wall and intense contrast enhancement resembling colitis with mesenteritis (Fig. 1).

**Figure 2:**
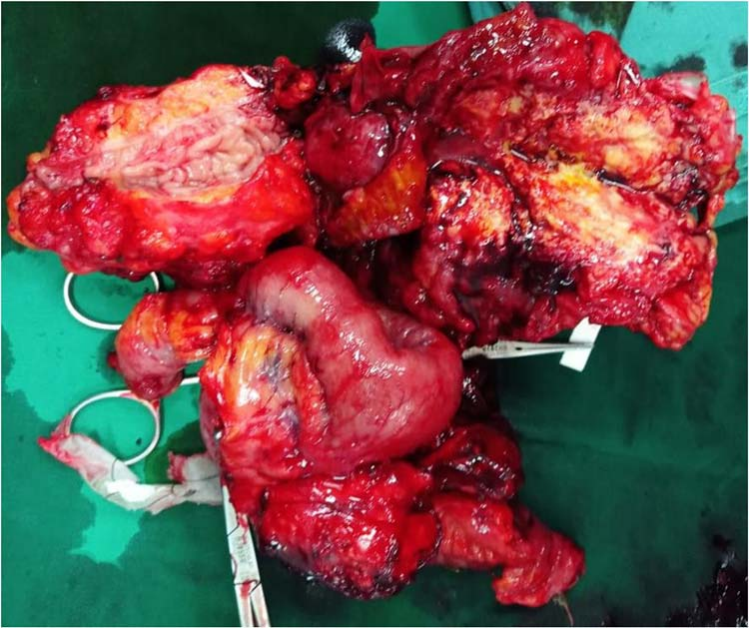
Intraoperative exploratory laparotomy situs: dense adhesional mass involving the sigmoid colon, surrounding ileum, and anterior peritoneum in the left lower quadrant mimicking advanced stage neoplasm (pseudotumor).

The patient underwent an exploratory laparotomy. At laparotomy, we found a dense adhesional mass in the left lower quadrant involving sigmoid colon with adjacent small bowel and peritoneum (Fig. 2). We decided to perform adhesiolysis between small bowel and sigmoid colon. The center of the adhesion was located on the extraluminal part of the intestine covering small bowel, transverse colon and sigmoid colon. During adhesiolysis the small bowel was completely lacerated at 150 and 170 cm from the Treitz ligament. The lacerated small bowel was resected and anastomosized end to end. On further exploration we discovered a mass (diameter 8 cm) in the outer wall of sigmoid colon. We decided to do the Hartmann procedure on sigmoid colon and exteriorize the proximal remaining part of sigmoid as an end colostomy.

**Figure 3:**
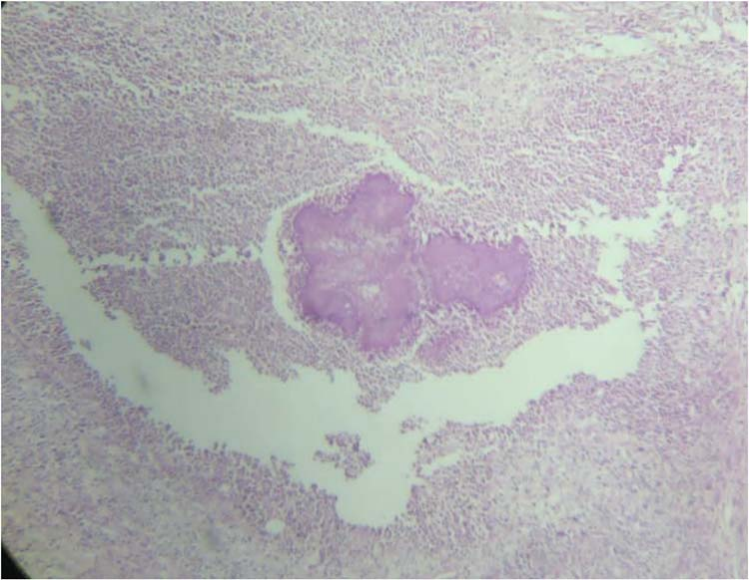
Sulfur granules appearance revealed from histopathological examination of sigmoid colon tissue which indicate actinomycose infection.

The biopsy of sigmoid colon revealed multiple nodules with abscess formation within the outer wall of bowel and focal diverticulitis. Microscopic findings showed characteristic sulfur granules of actinomycosis colony (Fig. 3). However, the culture result was negative. Intravenous antibiotic combination of 14-days ceftazidime and 5-days metronidazole was administered followed by complete resolution of symptoms.

## Discussion

Actinomyces species are nonmotile, filamentous, gram-positive, obligate anaerobic bacteria that commonly colonize the oral cavity, pharynx, gastrointestinal tract, genitourinary tract, and skin [6]. However, actinomyces can be an opportunistic infection in immunocompromised patient, called actinomycosis. In actinomycosis, pus production and abscess development are caused by a granulomatous inflammatory response, which is subsequently followed by necrosis and severe reactive fibrosis [7].

The incidence of actinomycosis generally is 1:300 000 with a male-to-female ratio of 3:1 and mainly occurs among patients between 20 and 60 years of age [7, 8]. Actinomycosis of the abdomen only occurs in 20% of cases, and involvement of the colon is very rarely reported [9, 10]. When the infection leaves the colon, it often only spreads locally; hematogenous or lymphatic dissemination are extremely rare [10]. In our case, the primary lesion started in the sigmoid colon with infiltration into the adjacent small bowel and peritoneum.

The clinical presentation of sigmoid colon actinomycosis is non-specific. Patients may present with chronic lower abdominal pain and pseudotumor abdominal mass or they may present with low-grade fever, acute abdominal pain, nausea, vomiting, constipation, or obstipation [11, 12].

Actinomycosis is very difficult to diagnose preoperatively (only about 10% of cases) [10]. Initially, the patient was suspected with perforated colon tumor based on the palpable mass, history of constipation and severe pain. The differential diagnosis was diverticulitis due to presentation of abdominal tenderness. The abdominopelvic type of intraabdominal actinomycosis is the most common type and typically results from impaired intestinal integrity, such as diverticulitis, gastrointestinal surgery, endoscopic procedures, or trauma. In the case of sigmoid colon actinomycosis, it can mimic diverticulitis or colon cancer [13]. Routine hematology and blood biochemistry rarely provide clues to this diagnosis. A simple abdominal radiograph can reveal intestinal obstruction and signs of perforation, which help clinical diagnosis [10]. A CT scan is helpful in identifying the inflammatory mass, site of the lesions, and the organs involved [14]. Bowel wall thickening and a homogeneous enhancement pattern are frequently found on contrast-enhanced CT scan. Some individuals may have pelvic or abdominal masses, which frequently manifest as an uneven enlargement [8]. In this case, CT scan with contrast showed thickening of the transverse, descending to sigmoid colon wall, surrounded by shadowy areas that enhance after contrast resembling colitis with mesenteritis.

Culture is the gold standard for diagnosis, but results are rarely positive because of the anaerobic characteristics and slow growth of Actinomyces. Cultures need to be processed within 15 minutes, put immediately into anaerobic conditions, and incubated for a lengthy period of time to demonstrate positive growth (from 5 to 20 days, and up to 76% of the cultures are negative) [9, 10, 15, 16]. Histopathological examination can be used to differentiate actinomycosis from neoplasms. The typical microscopic findings are necrosis with yellowish sulfur granules and filamentous Gram-positive fungal-like pathogens [10, 16, 17].

Actinomycosis has been detected by surgical biopsies in up to 71% of cases, proving the importance of surgical intervention in the diagnosis. The combination of surgery and antibiotics are effective in 90% of cases [17]. The long-term antibiotic regimen is needed with variables ranging from 3 weeks to 4 years [18]. Intravenous penicillin G for 2–6 weeks, followed by oral amoxicillin for 6–12 months is the first line therapy for actinomycosis [19]. A retrospective analysis of antimicrobial susceptibility testing of isolates showed that some antibiotics like carbapenems, tetracyclines, clindamycin, and vancomycin can be used as alternate therapies [10, 12, 20]. In this case, we used a third-generation cephalosporin (ceftazidime) and metronidazole because the time interval between surgery and histopathological results was about 7 days, and the administration of these antibiotics gave a positive response to the patient’s condition. Cephalosporins may be used to treat patients who are allergic to penicillin instead of aminopenicillins, although their clinical effectiveness is substantially lower than that of aminopenicillins [21].

## Conclusion

Histopathological and bacteriological examination are key to diagnose actinomycosis. Specific microscopic findings are yellowish sulfur granules. Patients require long-term antibiotic therapy, but surgery is often required because preoperative diagnosis is difficult. The combination of optimal surgical resection and antibiotics are effective in most cases and possibly reducing the duration of antibiotic therapy.
